# Chromosome-level genome assembly of a xerophytic plant, *Haloxylon ammodendron*

**DOI:** 10.1093/dnares/dsac006

**Published:** 2022-03-10

**Authors:** Mingcheng Wang, Lei Zhang, Shaofei Tong, Dechun Jiang, Zhixi Fu

**Affiliations:** 1 Institute for Advanced Study, Chengdu University, Chengdu 610106, China; 2 Key Laboratory of Ecological Protection of Agro-pastoral Ecotones in the Yellow River Basin, National Ethnic Affairs Commission of the People’s Republic of China, College of Biological Science & Engineering, North Minzu University, Yinchuan 750001, China; 3 MOE Key Laboratory for Bio-resources and Eco-environment, College of Life Science, Sichuan University, Chengdu 610105, China; 4 CAS Key Laboratory of Mountain Ecological Restoration and Bioresource Utilization & Ecological Restoration and Biodiversity Conservation Key Laboratory of Sichuan Province, Chengdu Institute of Biology, Chinese Academy of Sciences, Chengdu 610041, China; 5 College of Life Sciences, Sichuan Normal University, Chengdu 610101, China

**Keywords:** *Haloxylon ammodendron*, desert adaptation, xerophyte, PacBio’s high-fidelity sequencing, genome assembly

## Abstract

*Haloxylon ammodendron* is a xerophytic perennial shrub or small tree that has a high ecological value in anti-desertification due to its high tolerance to drought and salt stress. Here, we report a high-quality, chromosome-level genome assembly of *H. ammodendron* by integrating PacBio’s high-fidelity sequencing and Hi-C technology. The assembled genome size was 685.4 Mb, of which 99.6% was assigned to nine pseudochromosomes with a contig N50 value of 23.6 Mb. Evolutionary analysis showed that both the recent substantial amplification of long terminal repeat retrotransposons and tandem gene duplication may have contributed to its genome size expansion and arid adaptation. An ample amount of low-GC genes was closely related to functions that may contribute to the desert adaptation of *H. ammodendron*. Gene family clustering together with gene expression analysis identified differentially expressed genes that may play important roles in the direct response of *H. ammodendron* to water-deficit stress. We also identified several genes possibly related to the degraded scaly leaves and well-developed root system of *H. ammodendron*. The reference-level genome assembly presented here will provide a valuable genomic resource for studying the genome evolution of xerophytic plants, as well as for further genetic breeding studies of *H. ammodendron*.

## 1. Introduction

Desertification is a serious threat to arid and semi-arid regions, which cover more than 40% of the global land area and are home to about 1 billion people.^1^^,^^2^ This has become a major global crisis. Desertification has affected 10–20% of these drylands, and the desertification rate is likely to increase rapidly in the face of dramatic climate change and human activities.^3^^,^^4^ Deterioration of plant cover is the prime indicator of desertification. However, some plant species have developed their own adaptation strategies to overcome water and nutrient shortages in harsh desert environments.^5–8^ These extremophytes have played important roles in anti-desertification projects worldwide. Therefore, studying genetic mechanisms of plant adaptation to extreme desert environments will help us to develop more drought-resistant crops, which is beneficial for future desertification control. In the last few decades, molecular basis of drought and salt tolerance has been extensively studied in the model plant *Arabidopsis thaliana*.^9^ With the rapid development of sequencing technologies, our understanding of drought and salt stress response in plants has advanced by transcriptome analysis of a large number of xerophytic plants.^10^ However, genome sequencing of xerophytic plants, which is crucial for comprehensive understanding of their genetic mechanisms of adaptive evolution, has been limited to very few species, including one gymnosperm (*Welwitschia mirabilis*), one dicot species (*Ammopiptanthus nanus*), and three monocots (*Cleistogenes songorica*, *Oropetium thomaeum*, and *Aloe vera*).^11–15^

*Haloxylon ammodendron* (Amaranthaceae), a xerophytic perennial shrub or small tree with a high tolerance to drought and salt stress, dominates many sandy and saline areas of Asian deserts.^16^ It plays an important role in the maintenance of the structure and function of the desert ecosystem via sand fixation, wind control, and microclimate amelioration.^17^ In addition, this species has a high economic value because it has traditionally been used as livestock feed and firewood,^18^ and it is the host plant of a rare traditional Chinese medicinal plant, ‘desert ginseng’, *Cistanche deserticola*.^19^ A few xeromorphic and halomorphic characteristics have been developed in this species, including highly degraded scaly leaves, C_4_ photosynthesis in the succulent stem, a deep root system, and high salt content in the main stem and branch. To date, molecular-level studies of *H. ammodendron’*s response to abiotic stressors have been limited to the transcriptome^20–23^ and a limited number of genes.^24–26^ A comprehensive understanding of the adaptive evolution of *H. ammodendron* has been hampered by the lack of genome information.

In this study, we assembled a chromosome-level genome assembly of *H. ammodendron* (2*n* = 2*x* = 18)^27^ with PacBio’s high-fidelity (HiFi) reads and Hi-C reads. Using this high-quality genome, we investigated the evolutionary history and molecular mechanisms underlying the desert adaptation of *H. ammodendron*. The genome assembly presented here will provide a valuable genomic resource for studying the genome evolution of xerophytic plants. This genome is also beneficial for further genetic breeding studies of this ecologically and economically important species.

## 2. Materials and methods

### 2.1. Plant materials and sequencing

**Figure 1:**
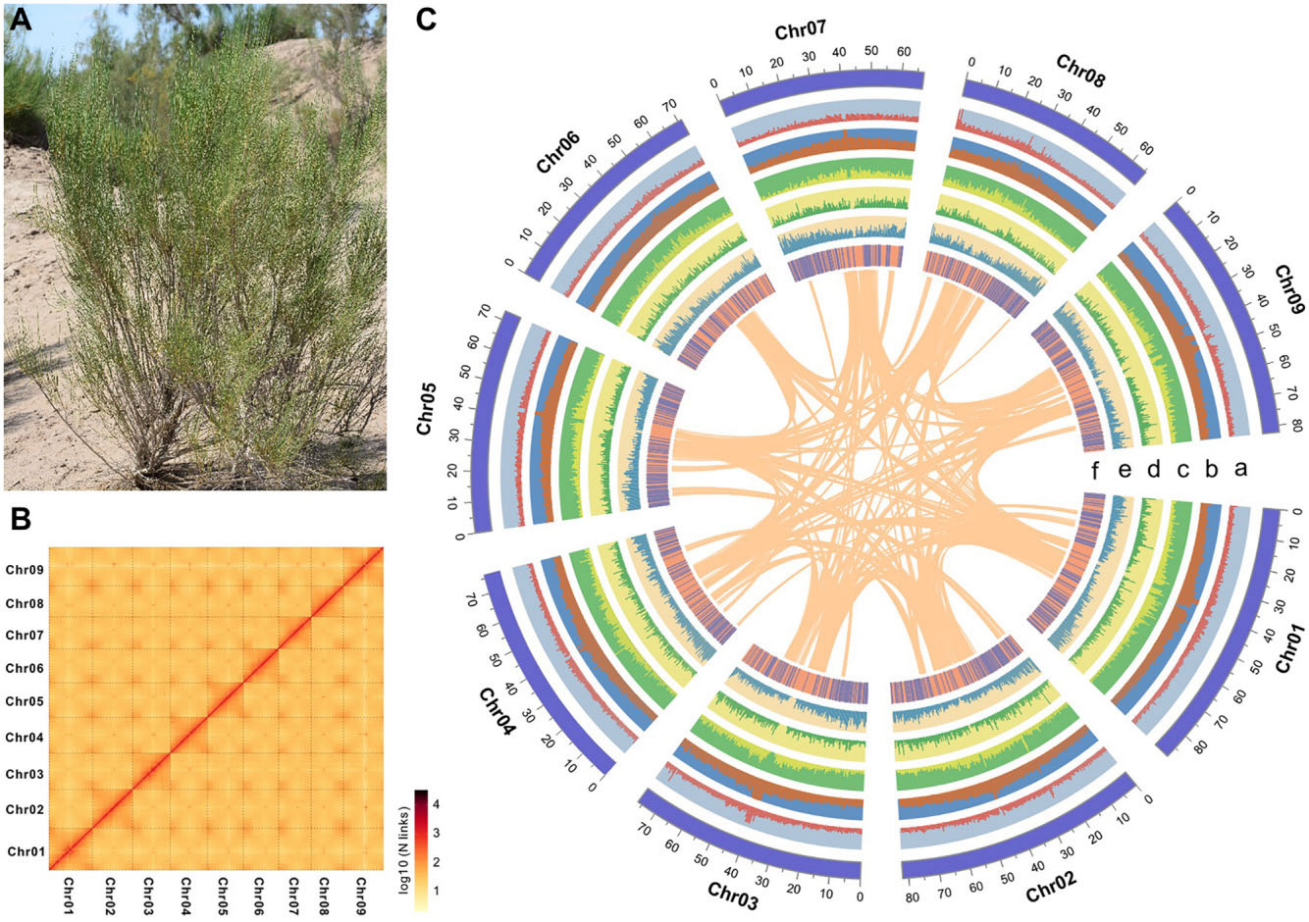
Overview of *H. ammodendron* and its genome. (A) Photo of an adult plant of *H. ammodendron*. (B) Heatmap showing Hi–C interactions at a resolution of 500 kb. (C) Landscape of the *H. ammodendron* genome. The tracks from outer to inner circles indicate the following: (a) GC content; (b) repeat density; (c) density of *Gypsy* elements; (d) density of *Copia* elements; (e) gene density; and (f) location of TF genes. The inner circle represents the collinear blocks identified in the *H. ammodendron* genome.

All plant materials used in this study were harvested from an adult plant of *H. ammodendron* growing in Yinchuan, Ningxia Province, northwestern China (Fig. 1A). The plant samples were authenticated by Dr Lei Zhang from North Minzu University, China. For whole-genome DNA sequencing, total genomic DNA was extracted from fresh leaves using the cetyl trimethylammonium bromide method.^28^ First, paired-end libraries with an insert size of 350 bp were constructed and sequenced on an Illumina HiSeq 2500 platform following the manufacturer’s instructions (Illumina, San Diego, CA, USA). Second, SMRTbell libraries were constructed using the PacBio 15-kb protocol (Pacific Biosciences, CA, USA), including DNA fragmentation, DNA damage repair and end repair, hairpin adapter ligation, and DNA purification. Long-read HiFi sequencing was then performed using circular consensus sequencing (CCS) mode on a PacBio Sequel II platform. Third, more than 2 g of young leaves were collected for the Hi-C experiment. Hi-C libraries were constructed as described previously,^29^ including chromatin extraction and digestion, DNA ligation, and purification. The DNA was sheared to a mean fragment size of ∼350 bp and sequenced on an Illumina HiSeq X Ten platform.

To aid in genome quality assessment and gene prediction, fresh leaves and stems from the same individual of *H. ammodendron* were collected for RNA sequencing (RNA-seq). Total RNA was extracted from these tissues, and a DNA-free DNA removal kit (Thermo Fisher Scientific, USA) was used to remove residual DNA. The RNA-seq libraries were prepared and sequenced on an Illumina HiSeq 2500 platform. RNA-seq reads were filtered using Trimmomatic v0.36^30^ with default parameters.

### 2.2. Genome assembly and assessment

The genome size of *H. ammodendron* was estimated based on a 17-mer frequency distribution of Illumina short reads generated by Jellyfish v2.2.9^31^ with parameters ‘-m 17 -s 3G -C’. Low-frequency *K*-mers (<4×) were removed and the genome size was calculated based on the formula Genome_size = (Total number of *k*-mers−Number of low-frequency *k*-mers)/Homozygous peak depth. Raw HiFi-sequencing data were processed using CCS v4.2.0 (https://ccs.how), with the main parameters of min-passes = 3 and min-rq = 0.99. The high-quality HiFi reads were then assembled into contigs using hifiasm v0.14,^32^ with default parameters. Further, ALLHiC^33^ was used to anchor these contigs into pseudochromosomes based on Hi-C data. Placement and orientation errors exhibiting obvious discrete chromatin interaction patterns were manually adjusted according to the contact maps plotted by Juicebox v1.8.8.^34^

Genome quality and completeness were assessed by a combination of short reads mapping, transcript alignment, benchmarking universal single-copy orthologs (BUSCO) analysis,^35^ and LTR assembly index (LAI) scores.^36^ First, the Illumina reads used in genome size estimation were mapped to the assembly using BWA v0.7.17,^37^ and the read depth distribution was calculated by SAMTOOLS v1.5.^38^ Second, RNA-seq reads were assembled into unigenes by Trinity v2.8.4^39^ under the mode without reference, and all unigenes were mapped to the genome using BLAT v36.^40^ Third, a BUSCO completeness score was calculated using BUSCO v3.02 in genome mode with the Embryophyta odb10 dataset. Finally, LAI scores were calculated in a sliding window of 3 Mb with a 300 kb step size across the entire genome using LTR_retriever v2.8.^41^

### 2.3. Genome annotation

The genome of *H. ammodendron* was annotated using procedures similar to those used in two previous studies.^42^^,^^43^ Homology searches of repeats were performed against the assembly by RepeatMasker v4.0.7^44^ based on a comprehensive database, including a *de novo* repeat library generated by RepeatModeler v1.0.11^45^ and the ‘Viridiplantae’ repeat library from the Repbase database v22.11.^46^ We also performed *de novo* detection of intact long terminal repeat retrotransposons (LTR-RTs) by LTR_Finder v1.06^47^ and LTRharvest v1.5.10,^48^ with the main parameters of min LTR length = 100 bp, max LTR length = 7,000bp, and min LTR similarity = 90%. Finally, LTR_retriever was used to integrate the LTR predictions, remove false positives, and estimate the insertion times of intact LTR-RTs. We then predicted protein-coding genes based on the repeat-masked genome. First, a homology-based prediction was conducted by aligning the protein sequences of *Suaeda aralocaspica* v1.0,^49^*Beta vulgaris* EL10_1.0,^50^*Chenopodium quinoa* v1.0,^51^*Spinacia oleracea* Spov3,^52^*Amaranthus hypochondriacus* v2.1,^53^ and *A.thaliana* TAIR10^54^ to the *H. ammodendron* genome with TBLASTN v2.2.31+^55^; GeneWise v2.4.1^56^ was used to predict gene structures based on the alignments. Second, Program to Assemble Spliced Alignment (PASA) v2.3.3^57^ was used to detect likely protein-coding regions based on a comprehensive transcriptome database comprising, *de novo* and genome-guided RNA-seq assembly. Third, a *de novo* prediction was performed using Augustus v3.2.3^58^ with parameters trained with high-quality gene models selected from the PASA predictions with an exon number ≥3 and CDS length ≥1,500bp. Finally, all gene predictions were integrated into a final gene set using EvidenceModeler (EVM) v1.1.1.^59^ The final gene set was searched against PlantTFDB v5.0^60^ to identify transcription factor (TF) genes.

Functional annotation was performed by aligning the protein-coding genes against the SwissProt and TrEMBL databases^61^ using DIAMOND v0.9.22.^62^ Protein motifs and domains were annotated using InterProScan v5.31^63^ by searching multiple publicly available databases. Gene ontology (GO) IDs for each gene were retrieved with Blast2GO v2.5.^64^ Kyoto Encyclopaedia of Genes and Genomes (KEGG) pathway mapping was achieved using the KEGG Automatic Annotation Server (https://www.genome.jp/kaas-bin/kaas_main).

### 2.4. Comparative genomic analysis

The protein sequences of *H. ammodendron* and seven other sequenced plant species, namely six Amaranthaceae species (*S.aralocaspica*, *B.vulgaris*, *Beta patula*,^65^*C.quinoa*, *S.oleracea*, and *A.hypochondriacus*) and one outgroup species (*A.thaliana*), were used for the phylogenetic analysis. Single-copy orthogroups among the eight species were distinguished using OrthoFinder v2.3.11^66^ with the DIAMOND aligner and the Markov cluster algorithm. For each single-copy orthogroup, protein sequences were aligned by MAFFT-LINSI v7.313.^67^ Conserved sites were extracted from the concatenated alignments of all single-copy genes using Gblocks v0.91b.^68^ A maximum likelihood phylogeny was constructed based on the conserved sites using RAxML v8.0.17^69^ under the PROTGAMMAILGX model with 100 bootstrap replicates.

Divergence times among the eight species were estimated using MCMCTREE implemented in PAML v4.9e^70^ with the options ‘independent rates’ and ‘F84’ models. The calibration points for the *A.thaliana*–*A.hypochondriacus* divergence (111–131 million years ago, Mya) and *S.oleracea*–*C.quinoa* divergence (18–57 Mya) were obtained from the TimeTree database (http://www.timetree.org). Gene family expansions and contractions were further determined using CAFE v3.1.^71^

To investigate gene families that are possibly involved in desert adaptation in *H. ammodendron* and xerophytes, gene family clustering was performed using OrthoFinder based on the protein sequences of *H. ammodendron*, *S.oleracea*, *A.thaliana*, *C.songorica*, and *O.thomaeum*. Gene families specific to xerophytes (*H. ammodendron*, *C. songorica*, and *O. thomaeum*) and specific to *H. ammodendron* were extracted using an in-house python script.

### 2.5. Whole-genome and tandem gene duplication analysis

The protein sequences of *H. ammodendron* and *S.aralocaspica* were self-aligned and aligned with each other using DIAMOND. Syntenic blocks within and between genomes were identified using MCScanX v1.1,^72^ with default parameters. For each syntenic gene pair, the non-synonymous substitution rate (*Ka*) and synonymous substitution rate (*Ks*) were calculated using the ‘add_ka_and_ks_to_collinearity.pl’ script from MCScanX. The prevalence of whole genome duplication (WGD) events was examined by checking the *Ks* distributions of paralogs and orthologs within and between the two species. A *Ka*/*Ks* ratio >1 indicates a positive selection.

Tandem gene duplications were identified as homologous gene pairs that (i) are located within 10 consecutive genes on the same pseudochromosome; (ii) are located within 100 kb of each other; and (iii) have an identity ≥50% and a coverage ≥70% obtained by BLASTP.

### 2.6. Gene expression analysis

Previously published RNA-seq data of 5% PEG-6000 (Osmotin mimics drought stress) treated *H. ammodendron* seedlings and controls^22^ were downloaded from the NCBI database under the BioProject PRJNA312463. Both the drought-treated seedlings and the controls had three biological replicates. RNA-seq reads were filtered by Trimmomatic and mapped to the assembly using HISAT2 v2.1.0.^73^ Fragments per kilobase per million (FPKM) was calculated to evaluate the gene expression level using Cufflinks v2.2.1.^74^ Genes that exhibited >2-fold change in FPKM values between drought-treated seedlings and controls were identified as up- or down-regulated genes.

## 3. Results and discussion

### 3.1. High-quality genome assembly of *H. ammodendron*

Before assembly, a total of 49.4 Gb Illumina data were generated and subjected to genome size estimation (Supplementary Table S1). In total, 44,091,563,459 *k*-mers (*k* = 17) were identified, and a major peak was observed around a *k*-depth of 61 (Supplementary Fig. S1). After removing low-frequency *k*-mers, the genome size of *H. ammodendron* was estimated to be 708.7 Mb, with a high degree of heterozygosity (1.04%).

To generate a high-accuracy and high-continuity genome assembly, we obtained a total of 22.6 Gb (31.9× genome coverage) HiFi long reads, with a maximum and average read length of 64.7 and 15.8 kb, respectively (Supplementary Table S1 and Fig. S2). *De novo* assembly of these HiFi reads resulted in a preliminary assembly comprising 132 contigs for a total length of 685.3 Mb (96.7% of the estimated genome size). Contigs longer than 1 Mb totalled 675.0 Mb (98.5% of the total assembly length), indicating the high continuity of the assembly. Coverage depth distribution of Illumina reads revealed that there were very few duplicate haplotypes in the assembly (Supplementary Fig. S3).

To reconstruct a chromosome-level assembly, we generated 84.7 Gb (119.6× genome coverage) Hi-C clean reads (Supplementary Table S1) and anchored the contigs into pseudochromosomes using the Hi-C scaffolding approach. Ultimately, 682.3 Mb sequences (99.6% of the entire assembly) were assigned to nine pseudochromosomes (Fig. 1B and C), corresponding to the haploid chromosome number of *H. ammodendron*. The lengths of pseudochromosomes ranged from 65.5 to 88.7 Mb (Supplementary Table S2), and all pseudochromosomes showed a well-organized diagonal pattern for intra-chromosomal interactions (Supplementary Fig. S4). The final genome assembly contained very few gaps, with an average gap number of 7.2 per pseudochromosome. The contig and scaffold N50 values of this assembly reached 23.6 and 76.2 Mb, respectively (Table 1 and Supplementary Table S3).

**Table 1:** Global statistics of *H. ammodendron* genome assembly and annotation

Assembly	
Estimated genome size (by *k*-mer analysis) (Mb)	708.69
Length of genome assembly (Mb)	685.35
Number of pseudochromosomes	9
Total length of pseudochromosomes (Mb)	682.32
Scaffold N50 (Mb)	76.23
Contig N50 (Mb)	23.61
Longest contig (Mb)	45.81
Gap size (bp)	6,500
Annotation	
GC content (%)	35.38
Percentage of repeat sequences (%)	46.48
Number of protein-coding genes	41,647
Average gene length (bp)	3,997.3
Average coding sequence length (bp)	1,075.3
Average exon length (bp)	277.4
Average intron length (bp)	783.6
Functionally annotated genes	39,032

To validate the quality of the genome assembly, we first mapped the Illumina reads back to the assembly and obtained an overall mapping rate of 98.6% and a 10-fold minimum genome coverage of 90.4% (Supplementary Table S4). We also assembled the RNA-seq reads into 306,064 unigenes, 82.7% of which had a length coverage of >90% within a single scaffold (Supplementary Table S5). Furthermore, we observed a BUSCO completeness score of 97.8% and an average LAI score of 19.8 for the entire genome (Supplementary Table S6 and Fig. S5). This evidence together indicated that the *H. ammodendron* assembly attained a high level of quality and completeness.

### 3.2. Annotation of the *H. ammodendron* genome

We predicted 318.6 Mb of repetitive sequences representing 46.5% of the *H. ammodendron* genome (Supplementary Table S7). The putative repetitive elements were more likely to be enriched in the central regions of chromosomes (Supplementary Fig. S6). The percentage of repetitive sequences in *H. ammodendron* was comparable to that of three other Amaranthaceae species, *B.vulgaris* (42.3%), *A.hypochondriacus* (48.1%), and *S.aralocaspica* (38.4%), but was much lower than that of *S.oleracea* (74.4%) and *C.quinoa* (64.0%) (Supplementary Table S8). Transposable elements (TEs) were the most abundant repeat class, spanning 182.7 Mb or 26.7% of the genome, with LTR-RTs being dominant (132.6 Mb; 19.4% of the genome) (Supplementary Table S7). Nearly 70% of TEs had a divergence rate of <20%, indicating a recent burst of TE elements in the *H. ammodendron* genome (Supplementary Fig. S7). In addition, we identified 125.1 Mb (18.3% of the genome) of unclassified repetitive sequences, which might be *H. ammodendron*-specific.

After masking all repetitive sequences, we identified a total of 41,647 protein-coding genes, 97.2% (40,467) of which were located on the nine pseudochromosomes, with an overall gene density of 59.3 gene per Mb (Supplementary Table S2). A BUSCO completeness score of 94.6% was obtained at the gene model level, indicating the high completeness of gene annotation (Supplementary Table S4). Compared to other Amaranthaceae species, *H. ammodendron* had the shortest average transcript length, which was mainly caused by intron length reduction (Supplementary Table S9). Of the protein-coding genes, 39,032 (93.7%) were functionally annotated in at least one publicly available database, and 21,946 (52.7%) genes were assigned to GO terms (Supplementary Table S10). Among the protein-coding genes, 1,340 TF genes were predicted and classified into 56 distinct gene families (Supplementary Table S11).

### 3.3. Evolutionary history of *H. ammodendron*

**Figure 2:**
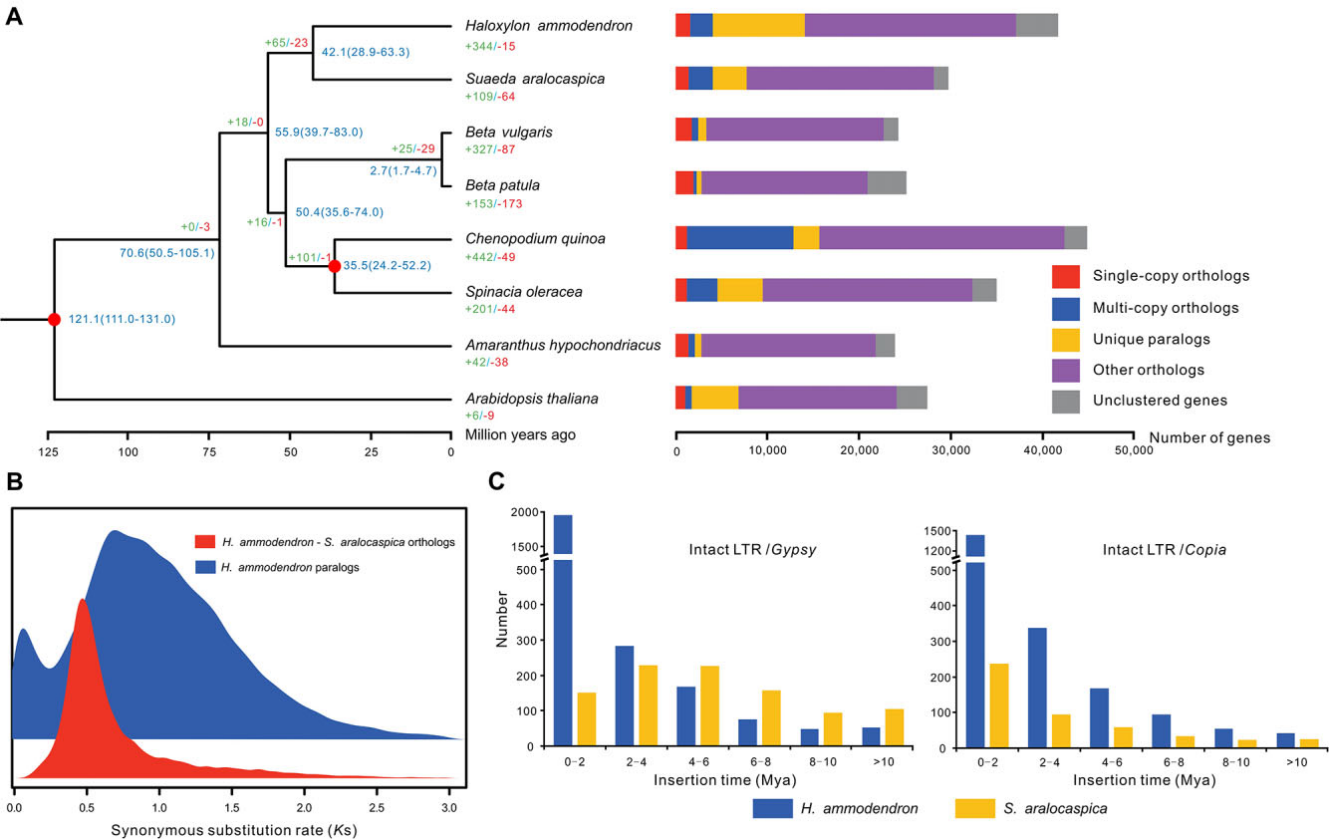
Phylogenetic and evolutionary analyses of the H. ammodendron genome. (A) A species tree based on 467 single-copy orthogroups from eight plant species with clusters of orthologous and paralogous gene families. Gene family expansion and contraction are denoted in the numbers next to the plus and minus signs, respectively. Numbers on nodes represent the inferred divergence times with 95% confidence intervals. Circles represent the calibration times obtained from the TimeTree database. (B) Ks distributions for the paralogs and orthologs identified from the whole genome of H. ammodendron and S. aralocaspica. (C) Insertion age distribution of H. ammodendron intact LTRs in comparison to S. aralocaspica.

We identified 467 strictly single-copy orthogroups among *H. ammodendron* and seven other plant species (Supplementary Table S12). A well-supported species tree was recovered based on the multiple sequence alignments of these single-copy genes (Fig. 2A). The results showed that *H. ammodendron* clustered together with another desert plant *S. aralocaspica* with 100% bootstrap support (Supplementary Fig. S8). This topology remained robust when we excluded the allotetraploid species *C. quinoa* from the phylogenetic analysis (Supplementary Fig. S8). The divergence time between *H. ammodendron* and *S. aralocaspica* was estimated to be around 42 Mya (Fig. 2A). However, the genome size of *H. ammodendron* (709 Mb) was significantly larger than that of *S. aralocaspica* (467 Mb). To gain further insights into the evolutionary history underlying genome size differences between these two species, we examined the prevalence of whole-genome/tandem duplication events and LTR amplifications, which were considered to be the major factors that contributing to genome size expansion.^75–77^ We identified a total of 31,220 collinear blocks within and between the genomes of *H. ammodendron* and *S. aralocaspica*. The *Ks* distribution of orthologs between these two species showed a major peak around 0.47, which is younger than the peak identified from the analysis of paralogs within *H. ammodendron* (∼0.69), indicating that no independent WGD event had occurred in the *H. ammodendron* genome after its split from *S. aralocaspica* (Fig. 2B). However, we found that the *H. ammodendron* genome displayed a high level of tandem duplication (2,343 arrays containing 7,245 genes), contrasting strongly with the *S. aralocaspica* genome (1,481 arrays containing 3,582 genes) (Supplementary Table S12). The *Ks* distribution of these duplicated genes suggests a recent burst of tandem duplication events in the *H. ammodendron* genome (Supplementary Fig. S9). Furthermore, we identified 6,357 intact LTR-RTs with a total length of 48.7 Mb (7.1% of the genome) in the *H. ammodendron* genome. In contrast, only 1,682 intact LTR-RTs with a total length of 13.0 Mb (2.9% of the genome) were identified in the *S. aralocaspica* genome. Approximately 52.9% of the intact LTR-RTs inserted into *H. ammodendron* were younger than 2 million years, with median insertion times of 1.02 and 0.56 Mya for *Copia* and *Gypsy* elements, respectively (Fig. 2C). Interestingly, the recent burst of LTR-RTs and gene duplications was coincided with the aridification process of Asian inland in late Cenozoic, which was possibly driven by the rapid uplift of the Tibetan Plateau or global cooling.^78^^,^^79^ These results indicate that both recent substantial amplification of LTR-RTs and tandem gene duplication in the *H. ammodendron* genome may have contributed to its genome size expansion and arid adaptation. In addition, we identified 344 significantly (*P* < 0.01) expanded gene families (containing 4,762 genes) in the *H. ammodendron* genome, which were functionally related to ‘defense response’, ‘response to water deprivation’, ‘meristem maintenance’, and ‘root development’ (Supplementary Fig. S10).

### 3.4. Molecular mechanisms underlying desert adaptation of *H. ammodendron*

**Figure 3:**
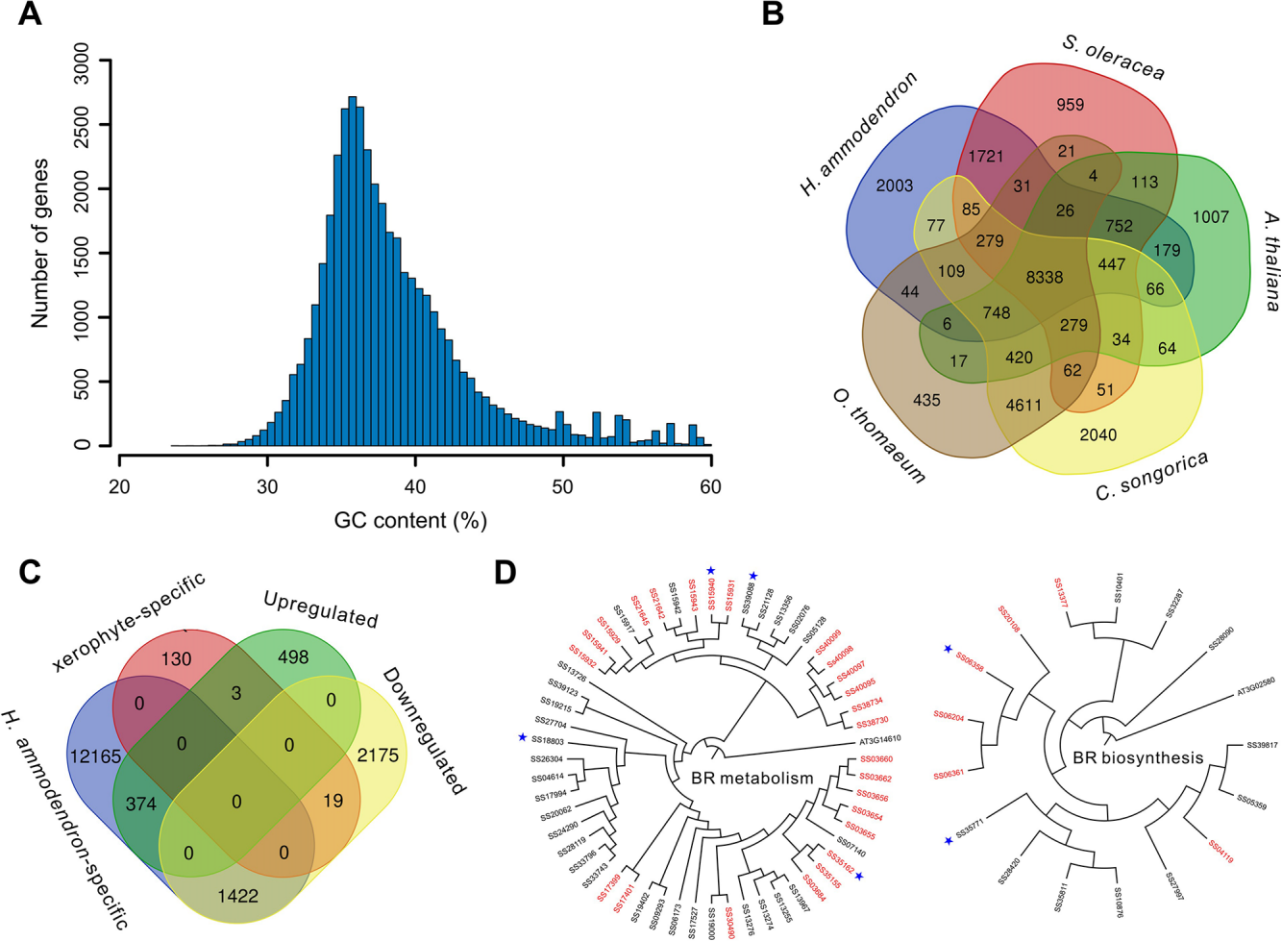
Molecular mechanisms underlying desert adaptation of H. ammodendron. (A) Histogram distribution of GC content of the protein-coding genes in the H. ammodendron genome. (B) Venn diagram of orthologous gene families identified among H. ammodendron, S. oleracea, A. thaliana, C. songorica, and O. thomaeum. (C) Venn diagram of xerophyte and H. ammodendron specific genes and up- and down-regulated genes in 5% PEG-6000 treated H. ammodendron seedlings compared with the controls. (D) Phylogenetic tree of genes involved in metabolism and biosynthesis of BRs in the H. ammodendron genome. Tandemly duplicated genes are highlighted, and positively selected genes are marked with pentagrams.

A recent study speculated that a low genomic GC content was possibly related to plant adaptation to harsh nutrient- and water-limited conditions.^11^ We observed a total GC content of 35.4% for the *H. ammodendron* genome, which was lower than that of non-exrophytic species in Amaranthaceae, such as *S. oleracea* (37.9%), *B. vulgaris* (35.9%), and *C. quinoa* (36.9%). The overall GC content of protein-coding genes in the *H. ammodendron* genome was 36.7%, with 1,669 genes exhibiting a GC content of <32.0% (Fig. 3A). These low-GC genes were found to be highly enriched in ‘seed growth’, ‘response to wounding’, ‘cellular response to osmotic stress’, ‘floral meristem determinacy’, ‘regulation of meristem development’, and ‘hyperosmotic salinity response’ (Supplementary Fig. S11). Reduction in the GC content of these genes may have contributed to the desert adaptation of *H. ammodendron*.

Furthermore, we performed gene family clustering between *H. ammodendron*, *S. oleracea*, *A. thaliana*, and two other xerophytic plants, *C.songorica* and *O.thomaeum*. A total of 36,670 genes in the *H. ammodendron* genome were assigned to 14,911 gene families. Among these gene families, 8,338 are common to all five species, whereas 109 are confined to xerophytic plants (*H. ammodendron*, *C. songorica*, and *O. thomaeum*) (Fig. 3B). The xerophyte-specific gene families were highly enriched in ‘response to heat’, ‘negative regulation of response to salt stress’, ‘positive regulation of abscisic acid biosynthetic process’, ‘stomatal closure’, ‘lateral root morphogenesis’, and ‘negative regulation of leaf development’ (Supplementary Fig. S12). We also identified 2,003 gene families (containing 13,961 genes) that are specific to *H. ammodendron* (Fig. 3B). These *H. ammodendron*-specific genes were markedly enriched in ‘response to water deprivation’, ‘embryo development ending in seed dormancy’, ‘response to wounding’, ‘circadian rhythm’, ‘response to brassinosteroid’, and ‘regulation of stomatal closure’ (Supplementary Fig. S13). Gene expression analysis identified 875 up-regulated and 3,616 down-regulated genes in 5% PEG-6000 treated *H. ammodendron* seedlings compared with the controls. Among these differentially expressed genes, 22 are specific to the three xerophytes, 1,796 are specific to *H. ammodendron* (Fig. 3C), 257 exhibit a GC content of <32.0%, and 839 are located in the significantly expanded gene families. These genes may play important roles in the direct response of *H. ammodendron* to water-deficit stress.

Desert plants usually develop smaller and narrower leaves than plants living in temperate habitat to limit water loss by transpiration, and obtain nutrients from soil through their well-developed roots for optimal growth.^8^ Brassinosteroids (BRs) are plant steroid hormones known mainly for promoting organ growth through their combined effect on cell expansion and division.^80^^,^^81^*Arabidopsis* mutant plants with known defects in the biosynthesis or perception of BRs develop small leaves.^82^ We identified 71 genes that were involved in metabolism (GO: 0016131) or biosynthesis (GO: 0016132) of BRs in the *H. ammodendron* genome (Fig. 3D and Supplementary Table S13). Among these genes, 31 were tandemly duplicated, and six were under positive selection (Fig. 3D). We also identified eight genes that were involved in the biosynthesis of strigolactones (SLs; GO: 1901601; Supplementary Table S13), which are a novel class of plant hormones controlling shoot branching in seed plants.^83^^,^^84^ In rice, treatment with SL biosynthesis inhibitor stopped elongation of seminal roots in wild-type plants under stress conditions.^85^ Based on Interproscan annotations, we identified 1 *SHORT-ROOT* (*SHR*), 1 *SHORT-ROOT-like* (*SHR-like*), 1 *SCARECROW* (*SCR*), and 19 *SCARECROW-like* (*SCR-like*) genes in the *H. ammodendron* genome (Supplementary Table S13), which may play important roles in the root development of *H. ammodendron*. Both the *SHR* and *SCR Arabidopsis* mutant plants showed dramatically shorter roots than wild-type.^86^^,^^87^*SHR-like* and *SCR-like* genes are also found to be closely linked to root formation in several plants.^88^^,^^89^ Among the genes identified above, nine BR metabolism/biosynthesis genes, one *SHR* gene, and two *SCR-like* genes were differentially expressed after drought treatment (Supplementary Table S13). These genes may contribute to the degraded scaly leaves and well-developed root system of *H. ammodendron*, which further promotes its adaptation to drought stress. These candidate genes provide essential information for future functional studies of the organ development of *H. ammodendron*.

## 4. Conclusion

In this study, we presented a chromosome-level genome assembly of *H. ammodendron* with high-level quality and completeness. We predicted 41,647 high-confidence protein-coding genes in the genome assembly. No clear evidence of a recent WGD event was found in the *H. ammodendron* genome. However, both the recent substantial amplification of LTR-RTs and tandem gene duplication may have contributed to its genome size expansion and arid adaptation. An ample amount of low-GC genes was closely related to functions that may contribute to the desert adaptation of *H. ammodendron*. Gene family clustering together with gene expression analysis identified differentially expressed genes that may play important roles in the direct response of *H. ammodendron* to water-deficit stress. We also identified several genes possibly related to the degraded scaly leaves and well-developed root system of *H. ammodendron*. The present high-quality reference genome sequence will accelerate desert adaptation studies of xerophytic plants as well as further genetic breeding studies of *H. ammodendron*.

## Supplementary data

Supplementary data are available at *DNARES* online.

## Funding

This work was supported by the National Natural Science Foundation of China (32070410, 32000158, and 31900322), the Sichuan Science and Technology Program (2020YFH0005), the Youth Innovation Promotion Association of CAS (2021370), the doctoral scientific research foundation of the North Minzu University (2021KYQD10), the Project of Sustainable Development Research Center of Resources and Environment of Western Sichuan, Sichuan Normal University (2020CXZX03), and start-up funds provided by Chengdu University (2081921039).

## Conflict of interest

None declared.

## Data availability

All sequence data have been deposited at the NCBI under the BioProject PRJNA786049. RNA-seq data are available under the Sequence Read Archive (SRA) accession numbers SRR17137407 and SRR17137408. Illumina short-read data, HiFi long-read data, and Hi-C data are available under the SRA accession numbers SRR17127859, SRR17129371, and SRR17138685. The genome assembly, annotations, and predicted peptides are available on FigShare at the link: https://doi.org/10.6084/m9.figshare.17128424.v1.

## Supplementary Material

dsac006_Supplementary_Data

## References

[1] Reynolds J.F. , Smith D.M.S., Lambin E.F., et al 2007, Global desertification: building a science for dryland development, Science, 316, 847–51. 17495163 10.1126/science.1131634

[2] Huang J. , Zhang G., Zhang Y., et al 2020, Global desertification vulnerability to climate change and human activities, Land Degrad. Dev., 31, 1380–91.

[3] Veron S.R. , Paruelo J.M., Oesterheld M. 2006, Assessing desertification, J. Arid Environ., 66, 751–63.

[4] Xu D. , Li C., Zhuang D., Pan J. 2011, Assessment of the relative role of climate change and human activities in desertification: a review, J. Geogr. Sci., 21, 926–36.

[5] Mulroy T.W. , Rundel P.W. 1977, Annual plants: adaptations to desert environments, Bioscience, 27, 109–14.

[6] Willert D.J. , Eller B.M., Werger M.J.A., Brinckmann E. 1990, Desert succulents and their life strategies, Vegetatio, 90, 133–43.

[7] Commander L.E. , Golos P.J., Miller B.P., Merritt D.J. 2017, Seed germination traits of desert perennials, Plant Ecol., 218, 1077–91.

[8] Kirschner G.K. , Xiao T.T., Blilou I. 2021, Rooting in the desert: a developmental overview on desert plants, Genes, 12, 709. 34068546 10.3390/genes12050709 PMC8151154

[9] Bartels D. , Sunkar R. 2005, Drought and salt tolerance in plants, Crit. Rev. Plant Sci., 24, 23–58.

[10] Ulrike B. 2018, Plant life in extreme environments: how do you improve drought tolerance? Front. Plant Sci., 9, 543. 29868044 10.3389/fpls.2018.00543 PMC5962824

[11] Wan T. , Liu Z., Leitch I.J. 2021, The Welwitschia genome reveals a unique biology underpinning extreme longevity in deserts, Nat. Commun., 12, 1–15. 34253727 10.1038/s41467-021-24528-4 PMC8275611

[12] Gao F. , Wang X., Li X., et al 2018, Long-read sequencing and *de novo* genome assembly of *Ammopiptanthus nanus*, a desert shrub, Gigascience, 7, giy074. 10.1093/gigascience/giy074 PMC6048559 29917074

[13] Zhang J. , Wu F., Yan Q., et al 2021, The genome of *Cleistogenes songorica* provides a blueprint for functional dissection of dimorphic flower differentiation and drought adaptability, Plant Biotechnol. J., 19, 532–47. 32964579 10.1111/pbi.13483 PMC7955882

[14] VanBuren R. , Bryant D., Edger P.P., et al 2015, Single-molecule sequencing of the desiccation-tolerant grass *Oropetium thomaeum*, Nature, 527, 508–11. 26560029 10.1038/nature15714

[15] Jaiswal S.K. , Mahajan S., Chakraborty A., Kumar S., Sharma V.K. 2021, The genome sequence of *Aloe vera* reveals adaptive evolution of drought tolerance mechanisms, Iscience, 24, 102079. 33644713 10.1016/j.isci.2021.102079 PMC7889978

[16] Huang Z. , Zhang X., Zheng G., Gutterman Y. 2003, Influence of light, temperature, salinity and storage on seed germination of *Haloxylon ammodendron*, J. Arid Environ., 55, 453–64.

[17] Ma Q. , Wang X., Chen F., et al 2021, Carbon sequestration of sand-fixing plantation of *Haloxylon ammodendron* in Shiyang River Basin: storage, rate and potential, Glob. Ecol. Conserv., 28, e01607.

[18] Song J. , Feng G., Tian C.Y., Zhang F.S. 2006, Osmotic adjustment traits of *Suaeda physophora*, *Haloxylon ammodendron* and *Haloxylon persicum* in field or controlled conditions, Plant Sci., 170, 113–9.

[19] Li X. , Zhang T.C., Qiao Q., et al 2013, Complete chloroplast genome sequence of holoparasite *Cistanche deserticola* (Orobanchaceae) reveals gene loss and horizontal gene transfer from its host *Haloxylon ammodendron* (Chenopodiaceae), PLoS One, 8, e58747. 23554920 10.1371/journal.pone.0058747 PMC3598846

[20] Long Y. , Zhang J., Tian X., et al 2014, *De novo* assembly of the desert tree *Haloxylon ammodendron* (CA Mey.) based on RNA-Seq data provides insight into drought response, gene discovery and marker identification, BMC Genomics, 15, 1–11. 25511667 10.1186/1471-2164-15-1111 PMC4377846

[21] Li Y. , Ma X., Zhao J., et al 2015, Developmental genetic mechanisms of C_4_ syndrome based on transcriptome analysis of C_3_ cotyledons and C_4_ assimilating shoots in *Haloxylon ammodendron*, PLoS One, 10, e0117175. 25643361 10.1371/journal.pone.0117175 PMC4313948

[22] Fan L. , Wang G., Hu W., et al 2018, Transcriptomic view of survival during early seedling growth of the extremophyte *Haloxylon ammodendron*, Plant Physiol. Biochem., 132, 475–89. 30292980 10.1016/j.plaphy.2018.09.024

[23] Gao H.J. , Lü X.P., Zhang L., et al 2017, Transcriptomic profiling and physiological analysis of *Haloxylon ammodendron* in response to osmotic stress, IJMS, 19, 84. 10.3390/ijms19010084 PMC5796034 29286291

[24] Wang B. , Du H., Yao Z., et al 2018, Validation of reference genes for accurate normalization of gene expression with quantitative real-time PCR in *Haloxylon ammodendron* under different abiotic stresses, Physiol. Mol. Biol. Plants, 24, 455–63. 29692553 10.1007/s12298-018-0520-9 PMC5911265

[25] Gao H. , Lü X., Ren W., et al 2020, HaASR1 gene cloned from a desert shrub, *Haloxylon ammodendron*, confers drought tolerance in transgenic *Arabidopsis thaliana*, Environ. Exp. Bot., 180, 104251.

[26] Gong L. , Zhang H., Liu X., et al 2020, Ectopic expression of HaNAC1, an ATAF transcription factor from *Haloxylon ammodendron*, improves growth and drought tolerance in transgenic Arabidopsis, Plant Physiol. Biochem., 151, 535–44. 32305820 10.1016/j.plaphy.2020.04.008

[27] Rice A. , Glick L., Abadi S., et al 2015, The chromosome counts database (CCDB)—a community resource of plant chromosome numbers, New Phytol., 206, 19–26. 25423910 10.1111/nph.13191

[28] Doyle J.J. , Doyle J.L. 1987, A rapid DNA isolation procedure for small quantities of fresh leaf tissue, Phytoch. Bull., 19, 11–5.

[29] Louwers M. , Splinter E., Van Driel R., De Laat W., Stam M. 2009, Studying physical chromatin interactions in plants using Chromosome Conformation Capture (3C), Nat. Protoc., 4, 1216–29. 19644461 10.1038/nprot.2009.113

[30] Bolger A.M. , Lohse M., Usadel B. 2014, Trimmomatic: a flexible trimmer for Illumina sequence data, Bioinformatics, 30, 2114–20. 24695404 10.1093/bioinformatics/btu170 PMC4103590

[31] Marçais G. , Kingsford C. 2011, A fast, lock-free approach for efficient parallel counting of occurrences of k-mers, Bioinformatics, 27, 764–70. 21217122 10.1093/bioinformatics/btr011 PMC3051319

[32] Cheng H. , Concepcion G.T., Feng X., et al 2021, Haplotype-resolved *de novo* assembly using phased assembly graphs with hifiasm, Nat. Methods, 18, 170–5. 33526886 10.1038/s41592-020-01056-5 PMC7961889

[33] Zhang X. , Zhang S., Zhao Q., et al 2019, Assembly of allele-aware, chromosomal-scale autopolyploid genomes based on Hi-C data, Nat. Plants, 5, 833–45. 31383970 10.1038/s41477-019-0487-8

[34] Durand N.C. , Shamim M.S., Machol I., et al 2016, Juicer provides a one-click system for analyzing loop-resolution Hi-C experiments, Cell Syst., 3, 95–8. 27467249 10.1016/j.cels.2016.07.002 PMC5846465

[35] Simão F.A. , Waterhouse R.M., Ioannidis P., Kriventseva E.V., Zdobnov E.M. 2015, BUSCO: assessing genome assembly and annotation completeness with single-copy orthologs, Bioinformatics, 31, 3210–2. 26059717 10.1093/bioinformatics/btv351

[36] Ou S. , Chen J., Jiang N. 2018, Assessing genome assembly quality using the LTR assembly index (LAI), Nucleic Acids Res., 46, e126. 30107434 10.1093/nar/gky730 PMC6265445

[37] Li H. , Durbin R. 2009, Fast and accurate short read alignment with Burrows–Wheeler transform, Bioinformatics, 25, 1754–60. 19451168 10.1093/bioinformatics/btp324 PMC2705234

[38] Li H. , Handsaker B., Wysoker A., et al; 1000 Genome Project Data Processing Subgroup. 2009, The sequence alignment/map format and SAMtools, Bioinformatics, 25, 2078–9. 19505943 10.1093/bioinformatics/btp352 PMC2723002

[39] Haas B.J. , Papanicolaou A., Yassour M., et al 2013, *De novo* transcript sequence reconstruction from RNA-seq using the Trinity platform for reference generation and analysis, Nat. Protoc., 8, 1494–512. 23845962 10.1038/nprot.2013.084 PMC3875132

[40] Kent W.J. 2002, BLAT—the BLAST-like alignment tool, Genome Res., 12, 656–64. 11932250 10.1101/gr.229202 PMC187518

[41] Ou S. , Jiang N. 2018, LTR_retriever: a highly accurate and sensitive program for identification of long terminal repeat retrotransposons, Plant Physiol., 176, 1410–22. 29233850 10.1104/pp.17.01310 PMC5813529

[42] Wang M. , Gu Z., Fu Z., Jiang D. 2021, High-quality genome assembly of an important biodiesel plant, *Euphorbia lathyris* L, DNA Res., 28, dsab022. 34664644 10.1093/dnares/dsab022 PMC8545615

[43] Wang M. , Tong S., Ma T., et al 2021, The chromosome-level genome assembly of Sichuan pepper provides insights into apomixis, drought tolerance, and alkaloid biosynthesis, Mol. Ecol. Resour., 21, 2533–45. 34145765 10.1111/1755-0998.13449

[44] Tarailo-Graovac M. , Chen N. 2009, Using RepeatMasker to identify repetitive elements in genomic sequences, Curr. Protoc. Bioinformatics, 25, 4–10. 10.1002/0471250953.bi0410s25 19274634

[45] Price A.L. , Jones N.C., Pevzner P.A. 2005, *De novo* identification of repeat families in large genomes, Bioinformatics, 21, i351–8. 15961478 10.1093/bioinformatics/bti1018

[46] Jurka J. , Kapitonov V.V., Pavlicek A., et al 2005, Repbase update, a database of eukaryotic repetitive elements, Cytogenet. Genome Res., 110, 462–7. 16093699 10.1159/000084979

[47] Xu Z. , Wang H. 2007, LTR_FINDER: an efficient tool for the prediction of full-length LTR retrotransposons, Nucleic Acids Res., 35, W265–8. 17485477 10.1093/nar/gkm286 PMC1933203

[48] Ellinghaus D. , Kurtz S., Willhoeft U. 2008, LTRharvest, an efficient and flexible software for *de novo* detection of LTR retrotransposons, BMC Bioinf., 9, 18. 10.1186/1471-2105-9-18 PMC2253517 18194517

[49] Wang L. , Ma G., Wang H., et al 2019, A draft genome assembly of halophyte *Suaeda aralocaspica*, a plant that performs C_4_ photosynthesis within individual cells, Gigascience, 8, giz116. 31513708 10.1093/gigascience/giz116 PMC6741815

[50] McGrath J.M. , Funk A., Galewski P., et al 2020, A contiguous *de novo* genome assembly of sugar beet EL10 (*Beta vulgaris* L.), bioRxiv, 10.1101/2020.09.15.298315. PMC9896481 36208288

[51] Jarvis D.E. , Ho Y.S., Lightfoot D.J., et al 2017, The genome of *Chenopodium quinoa*, Nature, 542, 307–12. 28178233 10.1038/nature21370

[52] Hulse-Kemp A.M. , Bostan H., Chen S., et al 2021, An anchored chromosome-scale genome assembly of spinach improves annotation and reveals extensive gene rearrangements in euasterids, Plant Genome, 14, e20101. 34109759 10.1002/tpg2.20101 PMC12806983

[53] Clouse J.W. , Adhikary D., Page J.T., et al 2016, The amaranth genome: genome, transcriptome, and physical map assembly, Plant Genome, 9, 1–14. 10.3835/plantgenome2015.07.0062 27898770

[54] Arabidopsis Genome Initiative. 2000, Analysis of the genome sequence of the flowering plant *Arabidopsis thaliana*, Nature, 408, 796–815. 11130711 10.1038/35048692

[55] Camacho C. , Coulouris G., Avagyan V., et al 2009, BLAST+: architecture and applications, BMC Bioinf., 10, 421. 10.1186/1471-2105-10-421 PMC2803857 20003500

[56] Birney E. , Clamp M., Durbin R. 2004, GeneWise and genomewise, Genome Res., 14, 988–95. 15123596 10.1101/gr.1865504 PMC479130

[57] Haas B.J. , Delcher A.L., Mount S.M., et al 2003, Improving the Arabidopsis genome annotation using maximal transcript alignment assemblies, Nucleic Acids Res., 31, 5654–66. 14500829 10.1093/nar/gkg770 PMC206470

[58] Stanke M. , Keller O., Gunduz I., et al 2006, AUGUSTUS: *ab initio* prediction of alternative transcripts, Nucleic Acids Res., 34, W435–9. 16845043 10.1093/nar/gkl200 PMC1538822

[59] Haas B.J. , Salzberg S.L., Zhu W., et al 2008, Automated eukaryotic gene structure annotation using EVidenceModeler and the program to assemble spliced alignments, Genome Biol., 9, R7. 18190707 10.1186/gb-2008-9-1-r7 PMC2395244

[60] Jin J. , Tian F., Yang D.C., et al 2016, PlantTFDB 4.0: toward a central hub for transcription factors and regulatory interactions in plants, Nucleic Acids Res., 45, D1040–5. 27924042 10.1093/nar/gkw982 PMC5210657

[61] Bairoch A. , Apweiler R. 2000, The SWISS-PROT protein sequence database and its supplement TrEMBL in 2000, Nucleic Acids Res., 28, 45–8. 10592178 10.1093/nar/28.1.45 PMC102476

[62] Buchfink B. , Xie C., Huson D.H. 2015, Fast and sensitive protein alignment using DIAMOND, Nat. Methods, 12, 59–60. 25402007 10.1038/nmeth.3176

[63] Hunter S. , Apweiler R., Attwood T.K., et al 2009, InterPro: the integrative protein signature database, Nucleic Acids Res., 37, D211–5. 18940856 10.1093/nar/gkn785 PMC2686546

[64] Conesa A. , Götz S., García-Gómez J.M., et al 2005, Blast2GO: a universal tool for annotation, visualization and analysis in functional genomics research, Bioinformatics, 21, 3674–6. 16081474 10.1093/bioinformatics/bti610

[65] Rodríguez del Río Á. , Minoche A.E., Zwickl N.F., et al 2019, Genomes of the wild beets *Beta patula* and *Beta vulgaris* ssp, Plant J., 99, 1242–53. 31104348 10.1111/tpj.14413 PMC9546096

[66] Emms D.M. , Kelly S. 2019, OrthoFinder: phylogenetic orthology inference for comparative genomics, Genome Biol., 20, 1–14. 31727128 10.1186/s13059-019-1832-y PMC6857279

[67] Katoh K. , Standley D.M. 2013, MAFFT multiple sequence alignment sofware version 7: improvements in performance and usability, Mol. Biol. Evol., 30, 772–80. 23329690 10.1093/molbev/mst010 PMC3603318

[68] Castresana J. 2000, Selection of conserved blocks from multiple alignments for their use in phylogenetic analysis, Mol. Biol. Evol., 17, 540–52. 10742046 10.1093/oxfordjournals.molbev.a026334

[69] Stamatakis A. 2014, RAxML version 8: a tool for phylogenetic analysis and post-analysis of large phylogenies, Bioinformatics, 30, 1312–3. 24451623 10.1093/bioinformatics/btu033 PMC3998144

[70] Yang Z. 2007, PAML 4: phylogenetic analysis by maximum likelihood, Mol. Biol. Evol., 24, 1586–91. 17483113 10.1093/molbev/msm088

[71] De Bie T. , Cristianini N., Demuth J.P., Hahn M.W. 2006, CAFE: a computational tool for the study of gene family evolution, Bioinformatics, 22, 1269–71. 16543274 10.1093/bioinformatics/btl097

[72] Wang Y. , Tang H., DeBarry J.D., et al 2012, MCScanX: a toolkit for detection and evolutionary analysis of gene synteny and collinearity, Nucleic Acids Res., 40, e49. 22217600 10.1093/nar/gkr1293 PMC3326336

[73] Kim D. , Langmead B., Salzberg S.L. 2015, HISAT: a fast spliced aligner with low memory requirements, Nat. Methods., 12, 357–60. 25751142 10.1038/nmeth.3317 PMC4655817

[74] Trapnell C. , Roberts A., Goff L., et al 2012, Differential gene and transcript expression analysis of RNA-seq experiments with TopHat and Cufflinks, Nat. Protoc., 7, 562–78. 22383036 10.1038/nprot.2012.016 PMC3334321

[75] Van de Peer Y. , Maere S., Meyer A. 2009, The evolutionary significance of ancient genome duplications, Nat. Rev. Genet., 10, 725–32. 19652647 10.1038/nrg2600

[76] Wendel J.F. , Jackson S.A., Meyers B.C., Wing R.A. 2016, Evolution of plant genome architecture, Genome Biol., 17, 1–14. 26926526 10.1186/s13059-016-0908-1 PMC4772531

[77] Qiao X. , Li Q., Yin H., et al 2019, Gene duplication and evolution in recurring polyploidization–diploidization cycles in plants, Genome Biol., 20, 1–23. 30791939 10.1186/s13059-019-1650-2 PMC6383267

[78] Lu H. , Wang X., Li L. 2010, Aeolian sediment evidence that global cooling has driven late Cenozoic stepwise aridification in central Asia, Geol. Soc. London Spec. Publ., 342, 29–44.

[79] Zhang Z. , Han W., Fang X., Song C., Li X. 2013, Late Miocene–Pleistocene aridification of Asian inland revealed by geochemical records of lacustrine-fan delta sediments from the western Tarim Basin, NW China, Palaeogeogr. Palaeoclimatol. Palaeoecol., 377, 52–61.

[80] Bajguz A. , Hayat S. 2009, Effects of brassinosteroids on the plant responses to environmental stresses, Plant Physiol. Biochem., 47, 1–8. 19010688 10.1016/j.plaphy.2008.10.002

[81] Gudesblat G.E. , Russinova E. 2011, Plants grow on brassinosteroids, Curr. Opin. Plant Biol., 14, 530–7. 21802346 10.1016/j.pbi.2011.05.004

[82] Nakaya M. , Tsukaya H., Murakami N., Kato M. 2002, Brassinosteroids control the proliferation of leaf cells of *Arabidopsis thaliana*, Plant Cell Physiol., 43, 239–44. 11867704 10.1093/pcp/pcf024

[83] Dun E.A. , Brewer P.B., Beveridge C.A. 2009, Strigolactones: discovery of the elusive shoot branching hormone, Trends Plant Sci., 14, 364–72. 19540149 10.1016/j.tplants.2009.04.003

[84] Brewer P.B. , Koltai H., Beveridge C.A. 2013, Diverse roles of strigolactones in plant development, Mol. Plant, 6, 18–28. 23155045 10.1093/mp/sss130

[85] Sun H. , Tao J., Gu P., Xu G., Zhang Y. 2016, The role of strigolactones in root development, Plant Signal. Behav., 11, e1110662. 26515106 10.1080/15592324.2015.1110662 PMC4871655

[86] Benfey P.N. , Linstead P.J., Roberts K., et al 1993, Root development in Arabidopsis: four mutants with dramatically altered root morphogenesis, Development, 119, 57–70. 8275864 10.1242/dev.119.Supplement.57

[87] Scheres B. , Di Laurenzio L., Willemsen V., et al 1995, Mutations affecting the radial organisation of the Arabidopsis root display specific defects throughout the embryonic axis, Development, 121, 53–62.

[88] Sanchez C. , Vielba J.M., Ferro E., et al 2007, Two SCARECROW-LIKE genes are induced in response to exogenous auxin in rooting-competent cuttings of distantly related forest species, Tree Physiol., 27, 1459–70. 17669736 10.1093/treephys/27.10.1459

[89] Sole A. , Sanchez C., Vielba J.M., et al 2008, Characterization and expression of a *Pinus radiata* putative ortholog to the Arabidopsis SHORT-ROOT gene, Tree Physiol., 28, 1629–39. 18765368 10.1093/treephys/28.11.1629

