# Supplementary material for: Chromosome-level genome assembly of a xerophytic plant, Haloxylon ammodendron
Source: DNA Res. 2022 Mar 10;29(2):dsac006. doi: 10.1093/dnares/dsac006 (PMC8946665; doi:10.1093/dnares/dsac006)
Supplement: dsac006_Supplementary_Data [file dsac006_supplementary_data.docx]

Supplementary materials for

**Chromosome-level genome assembly of a xerophytic plant, *Haloxylon ammodendron***

Mingcheng Wang^1#^, Lei Zhang^2#^, Shaofei Tong^3#^, Dechun Jiang^4,*^, Zhixi Fu^5,*^

^*^ Authors for correspondence: jiangdc@cib.ac.cn and fuzx2017@sicnu.edu.cn

| ***K*-mer** | ***K*-mer number** | **Peak depth** | **Genome size (Mb)** | **Used bases** | **Used reads** | **Heterozygous**  **Ratio (%)** |
| --- | --- | --- | --- | --- | --- | --- |
| 17 | 44,091,563,459 | 61 | 708.69 | 49,359,728,400 | 329,064,856 | 1.04 |

**Supplementary Figure S1. A 17-mer frequency distribution of *H. ammodendron* based on Illumina data.**

**Supplementary Figure S2. Plot of sub-reads length distribution of PacBio’s HiFi sequencing data.**

**Supplementary Figure S3. Read depth distributions on the *H. ammodendron* genome assembly.** The Illumina short reads were aligned onto the assembly using BWA; the number of aligned reads was then calculated for each base. This curve revealed that there were very few allelic haplotigs in the genome assembly.

**
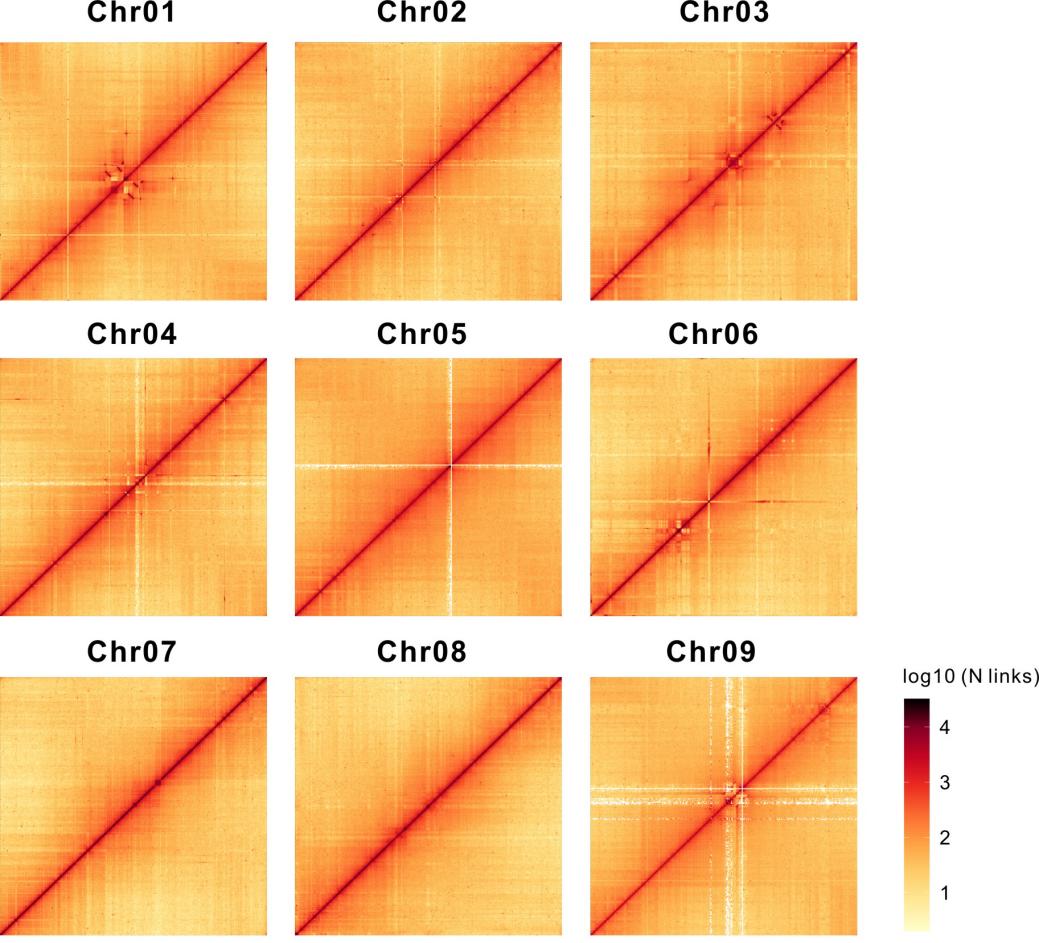
**

**Supplementary Figure S4. Heatmap showing Hi-C interactions of all *H. ammodendron* pseudochromosomes at a resolution of 100 kb.**

**Supplementary Figure S5. Distribution of LTR Assembly Index (LAI) score in the nine pseudo-chromosomes of *H. ammodendron* genome assembly.**

**Supplementary Figure S6. Distribution of repeat contents in the nine pseudo-chromosomes of *H. ammodendron* genome.** Each pseudo-chromosome was divided into ten bins of equal size.

**Supplementary Figure S7. Distribution of divergence rate of different transposable elements in the *H. ammodendron* genome.**

**Supplementary Figure S8. Phylogenetic relationship among *H. ammodendron* and other plant species (a) including and (b) not including the allotetraploid species *C. quinoa*.**

**Supplementary Figure S9. Histogram distribution of synonymous substitution rate (*Ks*) for gene pairs of tandem duplicates in *H. ammodendron*.**

**
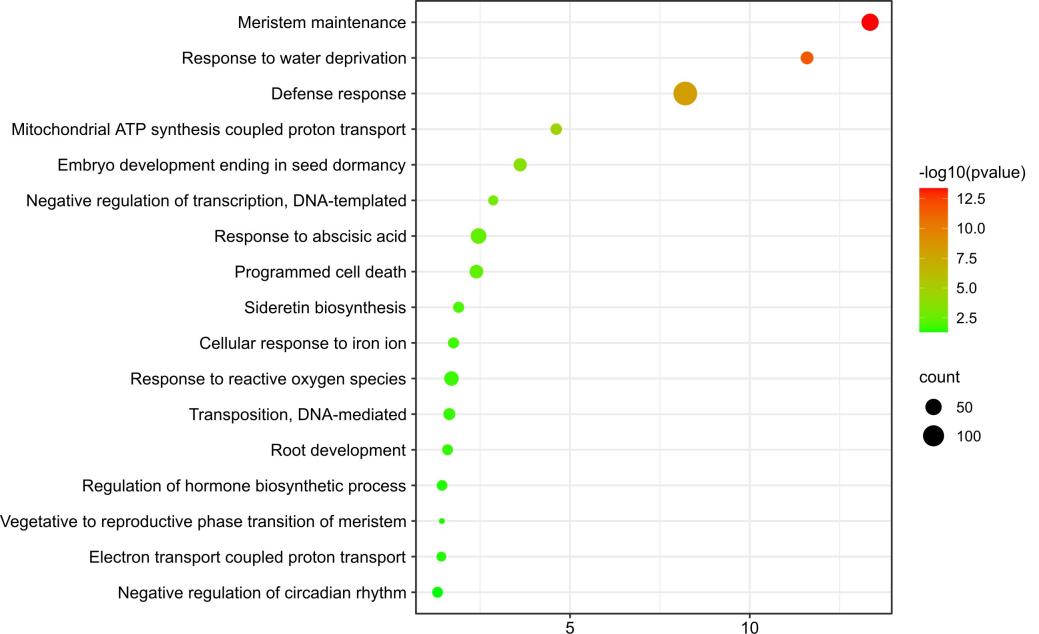
**

**Supplementary Figure S10. Enriched GO terms for the significantly expanded gene families of *H. ammodendron*.**

**Supplementary Figure S11. Enriched GO terms for the 1,669 low-GC genes in the *H. ammodendron* genome.**

**Supplementary Figure S12. Enriched GO terms for the xerophyte-specific gene families.**

**Supplementary Figure S13. Enriched GO terms for the *H. ammodendron*-specific gene families.**

| **Platform** | **Illumina** | **PacBio’s HiFi** | **Hi-C** |
| --- | --- | --- | --- |
| Read length (bp) | 150 | - | 150 |
| Number of reads | 164,532,428*2 | 1,429,136 | 282,417,015*2 |
| Number of bases (bp) | 49,359,728,400 | 22,596,680,507 | 84,725,104,500 |
| Sequence coverage (×)* | 69.65 | 31.89 | 119.55 |

* The estimated genome size was ~708.69 Mb.

**Supplementary Table S1.** **Summary of the Illumina, PacBio’s HiFi and Hi-C data for the assembly of *H. ammodendron* genome.**

| **Superscaffold** | **Length (bp)** | **Number of contigs** | **Number of genes** |
| --- | --- | --- | --- |
| Chr01 | 88,671,339 | 9 | 5,048 |
| Chr02 | 81,961,677 | 9 | 4,902 |
| Chr03 | 76,525,002 | 14 | 4,405 |
| Chr04 | 76,226,899 | 10 | 4,586 |
| Chr05 | 72,929,438 | 3 | 4,335 |
| Chr06 | 71,214,542 | 11 | 4,289 |
| Chr07 | 67,008,022 | 3 | 4,062 |
| Chr08 | 65,519,979 | 9 | 4,229 |
| Chr09 | 82,263,735 | 6 | 4,611 |
| **Total** | **682,320,633** | **74** | **40,467** |

**Supplementary Table S2.** **Summary of *H. ammodendron* superscaffolds.**

|  | **Contig** | | **Scaffold** | |
| --- | --- | --- | --- | --- |
|  | **Size (bp)** | **Number** | **Size (bp)** | **Number** |
| N90 | 9,221,544 | 27 | 65,519,979 | 9 |
| N80 | 14,945,733 | 21 | 71,214,542 | 7 |
| N70 | 19,030,989 | 17 | 71,214,542 | 7 |
| N60 | 21,151,614 | 13 | 72,929,438 | 6 |
| N50 | 23,613,282 | 10 | 76,226,899 | 5 |
| Longest | 45,814,771 | - | 88,671,339 | - |
| **Total** | **685,341,047** | **132** | **685,347,547** | **67** |

**Supplementary Table S3. Summary of the *H. ammodendron* genome assembly.**

|  |  | **Percentage (%)** |
| --- | --- | --- |
| Reads | Mapping rate (%) | 98.58 |
|  | Average sequencing depth | 59.04 |
|  | Coverage (%) | 95.02 |
| Genome | Coverage at least 4× (%) | 92.87 |
|  | Coverage at least 10× (%) | 90.38 |
|  | Coverage at least 20× (%) | 86.41 |

**Supplementary Table S4. Assessment of genome coverage rate using Illumina reads from 350 bp library.**

| **Dataset** | **Number** | **Total**  **length (bp)** | **Sequences covered by assembly (%)** | **With >90% sequence in one scaffold** | | **With >50% sequence in one scaffold** | |
| --- | --- | --- | --- | --- | --- | --- | --- |
|  |  |  |  | **Number** | **Percent (%)** | **Number** | **Percent (%)** |
| All | 306,064 | 327,342,683 | 95.65 | 253,199 | 82.73 | 288,112 | 94.13 |
| > 200 bp | 306,064 | 327,342,683 | 95.65 | 253,199 | 82.73 | 288,112 | 94.13 |
| > 500 bp | 182,025 | 287,398,743 | 98.76 | 155,267 | 85.30 | 177,257 | 97.38 |
| > 1000 bp | 114,456 | 238,933,208 | 99.62 | 98,435 | 86.00 | 112,645 | 98.42 |

**Supplementary Table S5. Evaluation of genome quality by alignment of transcripts assembled from RNA-seq reads.**

|  | **Assembly** | | **Annotation** | |
| --- | --- | --- | --- | --- |
|  | **Count** | **Ratio (%)** | **Count** | **Ratio (%)** |
| Complete BUSCOs | 1,579 | 97.83 | 1,527 | 94.61 |
| Complete and single-copy BUSCOs | 1,536 | 95.17 | 1,456 | 90.21 |
| Complete and duplicated BUSCOs | 43 | 2.66 | 71 | 4.40 |
| Fragmented BUSCOs | 9 | 0.56 | 31 | 1.92 |
| Missing BUSCOs | 26 | 1.61 | 56 | 3.47 |
| Total BUSCOs | 1,614 | 100.00 | 1,614 | 100.00 |

**Supplementary Table S6.** **The assessment of genome assembly and annotation completeness by BUSCO analysis.**

| **Type** | **Total length (bp)** | **% of genome** |
| --- | --- | --- |
| DNA | 30,882,278 | 4.51 |
| LINE | 19,038,202 | 2.78 |
| SINE | 135,674 | 0.02 |
| LTR | 132,629,847 | 19.35 |
| *Gypsy* | 94,372,428 | 13.77 |
| *Copia* | 35,003,018 | 5.11 |
| Other | 3,254,401 | 0.47 |
| Satellite | 412,484 | 0.06 |
| Simple repeat | 11,901,827 | 1.74 |
| Low complexity | 5,560 | 0.00 |
| Unknown | 125,139,820 | 18.26 |
| **Total** | **318,557,463** | **46.48** |

**Supplementary Table S7. Classification of repetitive elements in the *H. ammodendron* genome.**

| **Species** | **# of genes** | **Repeat content (%)** | **Version** | **Data source** |
| --- | --- | --- | --- | --- |
| *Suaeda aralocaspica* | 29,604 | 38.4 | v1.0 | GIGADB |
| *Beta vulgaris* | 24,255 | 42.3 | EL10_1.0 | Phytozome v13 |
| *Beta patula* | 25,068 | Not provided | v1.0 | The Sugar Beet Genome Project |
| *Chenopodium quinoa* | 44,776 | 64.0 | v1.0 | Phytozome v13 |
| *Spinacia oleracea* | 34,876 | 74.4 | Spov3 | Phytozome v13 |
| *Amaranthus hypochondriacus* | 23,847 | 48.1 | v2.1 | Phytozome v13 |
| *Arabidopsis thaliana* | 27,416 | 16.2 | TAIR10 | Phytozome v13 |

**Supplementary Table S8.** **Genome resources used for comparative genomics in this study.**

| **Species** | **Number of protein coding genes** | **Average gene length (bp)** | **Average CDS length (bp)** | **Average exon per gene** | **Average exon length (bp)** | **Average intron length (bp)** |
| --- | --- | --- | --- | --- | --- | --- |
| ***Haloxylon ammodendron*** | **41,647** | **3,997.26** | **1,075.30** | **4.73** | **227.39** | **783.61** |
| *Suaeda aralocaspica* | 29,604 | 4,261.99 | 1,117.70 | 4.76 | 235.03 | 837.22 |
| *Beta vulgaris* | 24,255 | 5,754.69 | 1,229.43 | 5.36 | 229.57 | 1,039.01 |
| *Beta patula* | 25,068 | 4,981.77 | 998.53 | 4.30 | 232.38 | 1,208.13 |
| *Chenopodium quinoa* | 44,776 | 4,552.02 | 1,274.44 | 5.45 | 233.71 | 736.03 |
| *Spinacia oleracea* | 34,876 | 4,244.22 | 1,207.26 | 4.90 | 246.63 | 779.70 |
| *Amaranthus hypochondriacus* | 23,847 | 4,515.19 | 1,067.39 | 4.87 | 219.13 | 890.67 |

**Supplementary Table S9. General statistics of predicted protein-coding genes in *H. ammodendron* and six other Amaranthaceae species.**

|  | **Number of genes** | **Percent (%)** |
| --- | --- | --- |
| Total | 41,647 | - |
| Annotated | 39,032 | 93.72 |
| InterPro | 37,988 | 91.21 |
| KEGG | 7,847 | 18.84 |
| SwissProt | 18,166 | 43.62 |
| TrEMBL | 32,044 | 76.94 |
| GO | 21,946 | 52.69 |
| Unannotated | 2,615 | 6.28 |

**Supplementary Table S10.** **Functional annotation of the protein-coding genes in the *H. ammodendron* genome assembly.**

| **TF family** | **Gene number** | **TF family** | **Gene number** | **TF family** | **Gene number** |
| --- | --- | --- | --- | --- | --- |
| AP2 | 13 | G2-like | 33 | NF-YA | 6 |
| ARF | 13 | GATA | 20 | NF-YB | 18 |
| ARR-B | 13 | GeBP | 2 | NF-YC | 7 |
| B3 | 84 | GRAS | 28 | Nin-like | 10 |
| BBR-BPC | 4 | GRF | 9 | RAV | 2 |
| BES1 | 7 | HB-other | 7 | SAP | 1 |
| bHLH | 117 | HB-PHD | 2 | SBP | 13 |
| bZIP | 47 | HD-ZIP | 26 | SRS | 5 |
| C2H2 | 74 | HRT-like | 3 | STAT | 1 |
| C3H | 48 | HSF | 13 | TALE | 13 |
| CAMTA | 4 | LBD | 35 | TCP | 14 |
| CO-like | 9 | LFY | 1 | Trihelix | 27 |
| CPP | 7 | LSD | 3 | VOZ | 2 |
| DBB | 4 | MIKC_MADS | 24 | Whirly | 4 |
| Dof | 21 | M-type_MADS | 35 | WOX | 11 |
| E2F/DP | 6 | MYB | 80 | WRKY | 60 |
| EIL | 4 | MYB_related | 53 | YABBY | 6 |
| ERF | 80 | NAC | 73 | ZF-HD | 9 |
| FAR1 | 127 | NF-X1 | 2 |  |  |

**Supplementary Table S11. Classification of transcription factor genes in the *H. ammodendron* genome.**

| **#Genes/array** | ***H. ammodendron*** | | ***S. aralocaspica*** | |
| --- | --- | --- | --- | --- |
|  | **#Array** | **#Gene** | **#Array** | **#Gene** |
| 2 | 1,428 | 2,856 | 1,123 | 2,246 |
| 3 | 435 | 1305 | 243 | 729 |
| 4 | 182 | 728 | 55 | 220 |
| 5 | 107 | 535 | 30 | 150 |
| 6 | 56 | 336 | 7 | 42 |
| 7 | 37 | 259 | 10 | 70 |
| 8 | 26 | 208 | 6 | 48 |
| 9 | 18 | 162 | 2 | 18 |
| 10 | 13 | 130 | 2 | 20 |
| >10 | 41 | 726 | 3 | 39 |
| Total | 2,343 | 7,245 | 1,481 | 3,582 |
| % of all genes | 17.4 | | 12.1 | |

**Supplementary Table S12. The tandemly duplicated gene arrays identified in the *H. ammodendron* and *S. aralocaspica* genome.**

| **Gene type** | **Candidate genes** |
| --- | --- |
| Brassinosteroid metabolism | *SS02076, SS03654,* ***SS03655*****, SS03656, SS03660, SS03662, SS03684, SS04614, SS05128, SS06173,* ***SS07140*****, SS09293, SS13255, SS13274, SS13276, SS13356, SS13726, SS13967, SS15917, SS15929, SS15931, SS15932, SS15940, SS15941,* ***SS15942*****, SS15943, SS17399, SS17401*, SS17527,* ***SS17994*****, SS18803, SS19000, SS19215, SS19402, SS20062, SS21128, SS21642, SS21645,* ***SS24290*****, SS26304, SS27704*, SS28119, SS30490, SS33743,* ***SS33796*****, SS35155, SS35162, SS38730, SS38734, SS39088, SS39123, SS40095, SS40097, SS40098, SS40099* |
| Brassinosteroid biosynthesis | *SS04119, SS05359, SS06204, SS06358, SS06361, SS10401, SS10876, SS13377, SS20108, SS27997, SS28090, SS28420, SS32287, SS35771, SS35811,* ***SS39817**** |
| Strigolactone biosynthesis | *SS00554*, *SS05910*, *SS15329*, *SS18123*, *SS22199*, *SS26610*, *SS28972*, *SS34720* |
| *SHORT-ROOT* | ***SS34780**** |
| *SHORT-ROOT-like* | *SS25057* |
| *SCARECROW* | *SS04208* |
| *SCARECROW-like* | *SS03139*, *SS05356*, *SS09895*, *SS11250*, *SS11250*, *SS11252*, *SS11252*, *SS11255*, *SS11257*, *SS24188*, ***SS27747****, *SS28244*, *SS28246*, *SS32690*, ***SS34750****, *SS34851*, *SS35745*, *SS36259*, *SS39934* |

**Supplementary Table S13. Information of genes possibly related to the degraded scaly leaves and well-developed root system of *H. ammodendron*. Genes differentially expressed after 5% PEG-6000 treatment were marked with asterisks.**
